# A case report focusing on diagnosis and intervention of chronic myeloid leukemia in blast crisis (acute megakaryoblastic leukemia subtype)

**DOI:** 10.3389/fonc.2025.1711432

**Published:** 2025-11-18

**Authors:** Long Liu, Yang Xiao, Na Cui, Liping Zhang, Lijing Wang, Cheng Gao, Jingfei Shi, Changyong Yuan, Chao Cui

**Affiliations:** 1 Department of Hematology, Qilu Hospital of Shandong University Dezhou Hospital, Dezhou, China; 2 General Practice, Qilu Hospital of Shandong University Dezhou Hospital, Dezhou, China; 3 School of Clinical Medicine, Shandong Second Medical University, Weifang, China; 4 Department of Clinical and Basic Medicine Shandong First Medical University, Jinan, Shandong, China

**Keywords:** chronic myeloid leukemia blast crisis, acute megakaryoblastic leukemia (M7), BCR/ABL1 fusion gene, orelabatinib, case report

## Abstract

**Objectives:**

To report a chronic myeloid leukemia (CML) blast crisis case, accurately diagnose its subtype, explore suitable treatment considering patient factors, and emphasize multidisciplinary diagnosis importance.

**Methods:**

Diagnosis relied on multiple approaches: bone marrow morphology, immunophenotyping, chromosomal karyotype analysis, and fusion gene detection to identify the subtype of CML blast crisis. Given the patient’s financial constraints and drug availability, the third - generation tyrosine kinase inhibitor (TKI) orelabatinib was chosen for treatment. Minimal residual disease (MRD) was rechecked two weeks after initiating therapy to assess treatment efficacy.

**Results:**

The patient was diagnosed with CML blast crisis of the acute megakaryoblastic leukemia subtype (M7), presenting with BCR/ABL1 fusion gene positivity, complex karyotypic abnormalities, and secondary myelofibrosis. After two weeks of orelabatinib treatment, MRD levels significantly declined, demonstrating the therapy’s effectiveness.

**Discussion:**

This case underscores the necessity of multidisciplinary collaboration for accurate diagnosis. Treatment selection for rare subtypes like M7 requires balancing medical need with patient - specific factors. The successful reduction in MRD validates the rationality of orelabatinib use, yet more research on treatment options for rare subtypes is warranted.

**Conclusion:**

Multidisciplinary methods are crucial for diagnosing CML blast crisis. Orelabatinib shows efficacy, and more research on personalized treatment is needed.

## 1 Introduction

Chronic myeloid leukemia (CML) is a myeloproliferative neoplasm characterized by the presence of the Philadelphia chromosome and the BCR/ABL1 fusion gene. The blast crisis phase, representing the most advanced stage of CML, is associated with a dismal prognosis, especially when it transforms into rare subtypes such as acute megakaryoblastic leukemia (AML-M7). While tyrosine kinase inhibitors (TKIs) have revolutionized the management of CML, patients in blast crisis often require more aggressive and individualized treatment strategies due to disease complexity and potential TKI resistance (1–3).

What makes this case truly unique is the combination of several rare features. Firstly, the presentation of CML blast crisis as AML-M7 is infrequent, accounting for only a small fraction of CML transformations. Secondly, the coexistence of complex chromosomal abnormalities and secondary myelofibrosis further complicates the diagnosis and treatment landscape. Additionally, the successful use of olverembatinib, a third-generation TKI, in a resource-constrained setting to achieve a rapid reduction in minimal residual disease highlights the importance of tailoring therapy to individual patient circumstances. This case not only underscores the significance of comprehensive diagnostic workup involving multiple modalities but also offers valuable insights into therapeutic decision-making for rare and challenging CML blast crisis presentations.

## 2 Case data

### 2.1 Basic information

The patient, Zhao XX, is a 54-year-old female clerk of Han ethnicity from Decheng District, Dezhou City, Shandong Province, China. She was first admitted to the hospital on July 22, 2023, due to “discovery of a left upper abdominal mass for more than 1 month”. She had a 9-year history of thyroid nodules and a 5-year history of breast hyperplasia, with no family history of genetic diseases.

### 2.2 Present illness

The patient noticed a palpable mass in the left upper quadrant of the abdomen without obvious precipitating factors approximately one month ago, accompanied by unintentional weight loss (approximately 3 kg). No gastrointestinal symptoms such as abdominal pain, diarrhea, acid reflux, or nausea were reported. One day prior to admission, outpatient laboratory tests revealed marked leukocytosis (17.88×10^9^/L) and splenomegaly (spleen type I line 12 cm and type II line 13 cm), prompting hospitalization for further diagnostic evaluation.

### 2.3 Physical examination

- Vital signs: Temperature 36.3 °C, pulse 78 beats/min, respiration 18 breaths/min, blood pressure 132/72 mmHg.
- Abdomen: Spleen palpable below the costal margin, firm in texture, smooth margin, no tenderness; liver not palpable.
- Others: Skin without pallor, jaundice, or petechiae; no palpable enlarged lymph nodes in bilateral neck, axilla, or inguinal regions.

## 3 Auxiliary examinations

### 3.1 Laboratory tests

**Figure 1:**
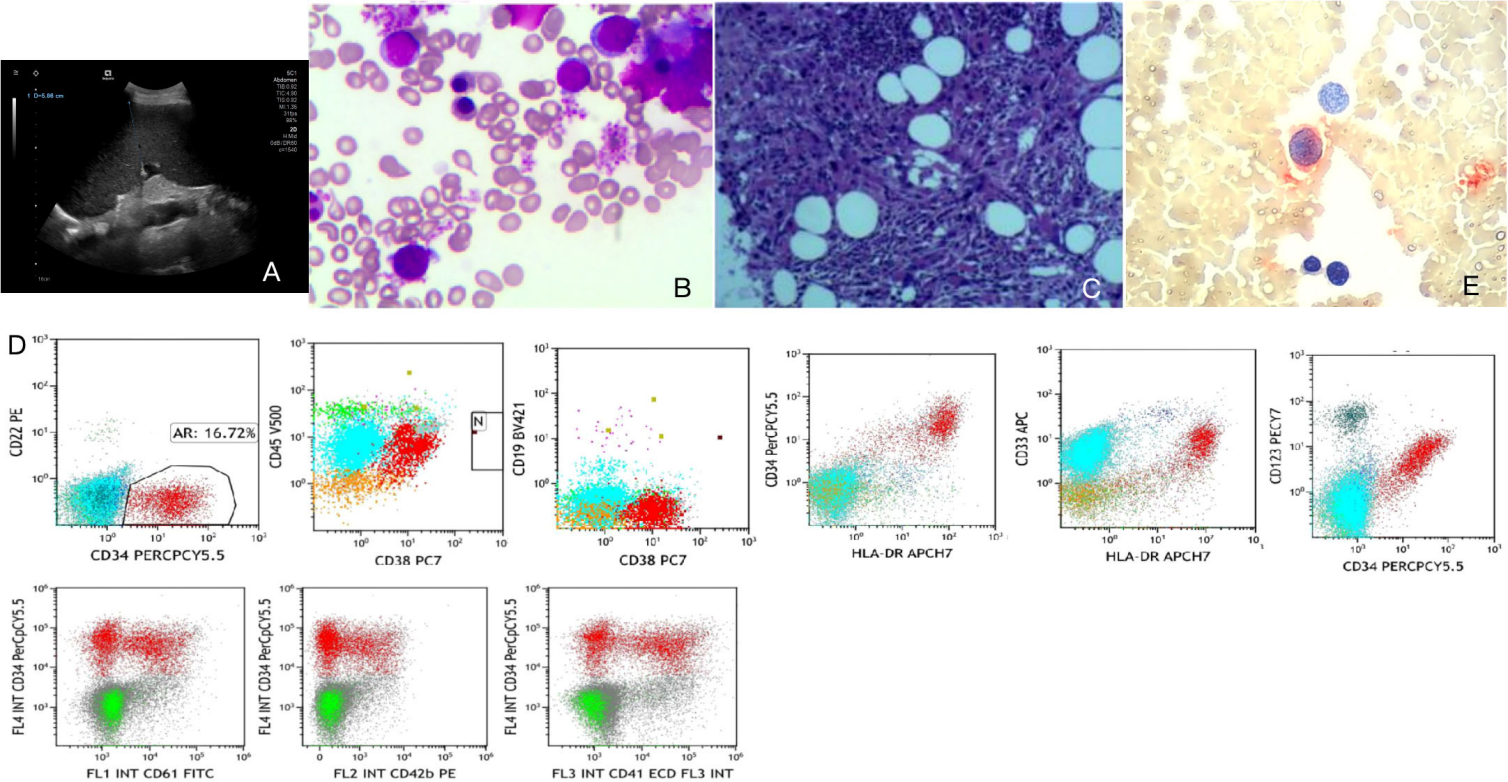
Auxiliary examinations. **(A)** Abdominal color Doppler ultrasound. **(B)** Bone marrow smear. **(C)** Bone marrow pathology. **(D)** Immunophenotyping. **(E)** Small megakaryocyte enzyme labeling.

- Blood routine: White blood cell count 17.88×10^9^/L (↑), hemoglobin 100 g/L (↓), platelet count 282×10^9^/L, C-reactive protein 3.64 mg/L, erythrocyte sedimentation rate 20 mm/h.
- Blood smear: Neutrophilic stab granulocytes 33%, juvenile granulocytes 9%, basophils 10%, indicating abnormal granulocytic hyperplasia.
- Abdominal color Doppler ultrasound: Splenomegaly (thickness 5.9 cm, length 19 cm), hepatic cysts, coarsened hepatic parenchymal echogenicity (**Figure 1A**).

### 3.2 Bone marrow examinations

- Bone marrow smear: Primitive cells accounted for 21.5%, with immature megakaryocytes and lymphoid small megakaryocytes observed. POX was negative, PAS was positive, suggesting a possibility of acute megakaryoblastic leukemia (M7) (**Figure 1B**).
- Bone marrow pathology: Dense hyperplasia of megakaryocytes with small cell bodies and few lobes; CD42b- and CD61-positive megakaryocytes, consistent with morphological features of CML blast crisis (**Figure 1C**).
- Immunophenotyping: Abnormal cell population accounted for 16.72%, expressing CD34, HLA-DR, CD38, CD33, and CD123; partial expression of CD117, CD41, and CD42b; negative for myeloid markers such as CD15, CD22, and MPO, supporting megakaryoblastic origin (**Figure 1D**; **Table 1**).
- Small megakaryocyte enzyme labeling: Approximately 50% of primitive cells showed positive or weakly positive staining, further indicating a tendency toward the M7 subtype (**Figure 1E**).

**Table 1:** Results of immunophenotyping.

Phenotype	Antigen
Expression	CD34, HLA-DR, CD38, CD33, CD123
Partial Expression	CD117, CD61, CD41, CD42b
Weak Expression	CD13, CD36, CD71, weak expression of CD7 in partial cells
No Expression	CD15, CD64, CD11b, CD22, CD5, CD2, CD20, CD19, CD10, CD4, CD14, MPO, CD9, TDT, cCD79a, cCD3, mCD3, CD56, CD105

### 3.3 Genetic examinations

**Figure 2:**
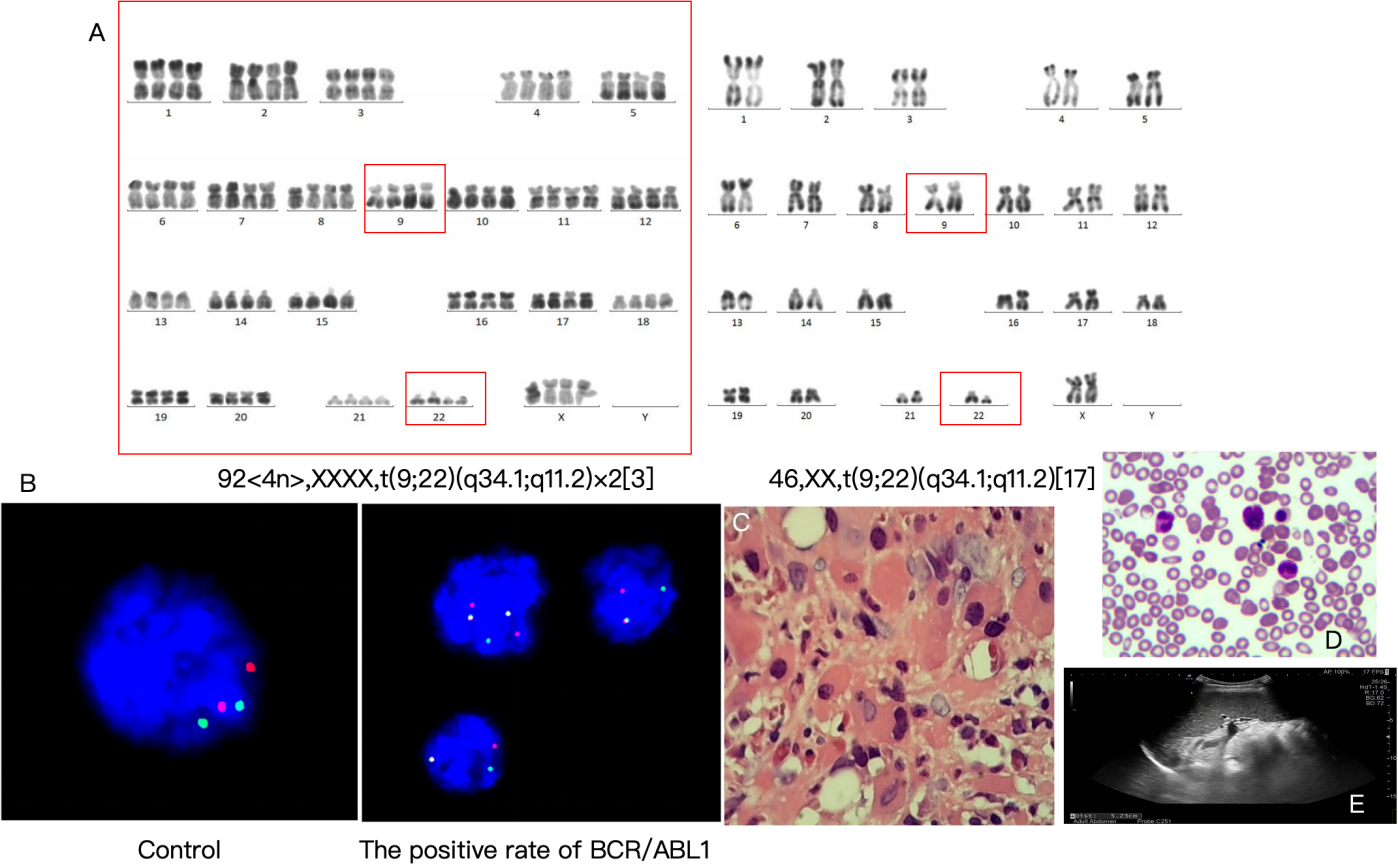
Genetic examinations, diagnostic results, and treatment outcomes. **(A)** Chromosomal karyotype. **(B)** Fusion gene. **(C)** Pathological consultation results. **(D)** Reexamination results on August 11, 2023 (2 weeks after treatment). **(E)** Abdominal color Doppler ultrasound (2 weeks after treatment).

- Chromosomal karyotype: 46,XX,t(9;22)(q34.1;q11.2)[17]/92,<4n>,XXXX,t(9;22)(q34.1;q11.2)×2[3], indicating Ph chromosome positivity and complex karyotypic abnormalities (**Figure 2A**).
- Fusion gene: BCR/ABL1 P210 positive (quantitative 61.5%), FISH detection positivity rate 96%, with positivity in mature granulocytes, megakaryocytes, eosinophils/basophils, confirming the diagnosis of CML blast crisis (**Figure 2B**).

## 4 Diagnosis and differential diagnosis

### 4.1 Diagnosis

- Chronic myeloid leukemia in blast crisis (acute megakaryoblastic leukemia subtype, M7) (**Figure 2C**).
- Secondary myelofibrosis (**Figure 2C**).

### 4.2 Differential diagnosis

- Primary acute myeloid leukemia (AML-M7): Acute megakaryoblastic leukemia without a history of CML needs to be excluded. However, the presence of BCR/ABL1 fusion gene and Ph chromosome in this case supports CML blast crisis.
- Myelodysplastic syndrome (MDS)/myeloproliferative neoplasm (MPN) overlap syndrome: Dysmorphic megakaryocytes and myelofibrosis require differentiation, but positive fusion gene findings rule out this diagnosis.

## 5 Treatment and follow-up

### 5.1 Treatment strategy

- Local drug availability: The BCR-ABL TKIs available in the region where this study was conducted are categorized by generation as follows: first-generation, imatinib mesylate; second-generation, including flumatinib, nilotinib, and dasatinib; and third-generation, olverembatinib.
- Basis for medication selection: The selection of olverembatinib for the patient in this study was based on three core considerations: 1) Urgency of the patient’s condition and prognostic needs: The patient had progressed to the blast crisis phase of CML. This phase is characterized by rapid disease progression and extremely poor prognosis, necessitating a highly potent therapeutic regimen to rapidly control disease progression and improve prognosis. 2) Clinical evidence supporting drug efficacy: Compared with first-generation TKIs (imatinib mesylate) and second-generation TKIs (flumatinib, nilotinib, dasatinib), olverembatinib, as a third-generation TKI, exhibits stronger inhibitory activity against the BCR-ABL fusion gene (especially for potential accompanying drug-resistant mutations) in patients with CML blast crisis. Clinical studies have confirmed that olverembatinib achieves superior hematologic response rates and molecular response rates in patients with CML blast crisis, and this efficacy advantage aligns with the patient’s therapeutic needs. 3) Practical constraints of local drug accessibility: As mentioned earlier, olverembatinib is the only accessible third-generation TKI in the region where this study was conducted, with no alternative drugs of the same generation available. From the perspective of clinical feasibility and timeliness of treatment, olverembatinib represents the optimal choice to meet the therapeutic needs of this patient with CML blast crisis.
- Targeted therapy: Due to limited financial resources, the patient was prescribed the third-generation TKI orelabatinib (40 mg, oral administration every other day), which is effective for patients with BCR/ABL1 T315I mutation resistance.
- Supportive care: Red blood cell transfusion was administered to correct anemia, with regular monitoring of blood routine and liver/kidney function.

### 5.2 Efficacy evaluation

- Reexamination on August 11, 2023 (2 weeks after treatment) showed: bone marrow primitive cells decreased to 1%, minimal residual disease (MRD) 1.66%, indicating treatment efficacy (**Figures 2D, E**; **Table 2**).
- Follow-ups were conducted at 1.5 months, 4 months, 8.5 months, and 10 months after treatment, with details listed in **Table 2**.
- Adverse and unanticipated events: Patients have limited financial resources, and long-term medication may lead to treatment interruption.

**Table 2:** Analysis of treatment efficacy.

Date	Treatment phase	Blood routine				Bone marrow smear	BCR/ABL(%)	Splenic color doppler ultrasound	Medication administration	Significance
		White blood cell (10^9^/L)	Neutrophil (10^9^/L)	Hemoglobin (g/L)	Platelet (10^9^/L)					
2023/7/22	Before Treatment	15.45	9.69	98	277	Hyperplasia is obviously active; abnormal hyperplasia of primitive cells accounts for 21.5%. Megakaryocytes are distributed in clusters throughout the slide, with single-round and double-round megakaryocytes observed. Immature megakaryocytes with platelet-producing phenomena and lymphoid small megakaryocytes are visible.	43.6666	The spleen is 5.9 cm in thickness and 19 cm in length.		FISH results show BCR/ABL positivity in neutrophils, indicating transformation of the patient to chronic myeloid leukemia.
2023/8/11	Two Weeks after Treatment	5.84	4.17	105	141	Hyperplasia is still active, with primitive granulocytes accounting for 1%.	42.8495	The thickness of the spleen is 5.2 cm, and the long diameter is 15 cm.	Orelabatinib (40mg qod)	Transition to chronic phase.
2023/9/16	1.5 Months after Treatment	3.39	1.51	118	88		9.6695		Orelabatinib (30mg qod)	BCR/ABL negativity indicates that the disease has achieved deep molecular remission.
2023/12/4	4 Months after Treatment	7.47	3.79	138	54					
2024/4/17	8.5 Months after Treatment	7.27	4.54	138	130		0			
2024/5/30	10 Months after Treatment	7.03	4.21	138	137					

## 6 Discussion

### 6.1 Rarity of M7 subtype in CML blast crisis

The M7 subtype accounts for <5% of CML blast crisis cases, with extremely poor prognosis and a median survival of only 3–6 months. This case was characterized by splenomegaly and myelofibrosis, prone to misdiagnosis as primary AML or MDS/MPN overlap syndrome, requiring confirmation by fusion gene and chromosomal karyotype analysis.

### 6.2 Morphological challenges in megakaryoblastic leukemia

The morphology of primitive megakaryocytes can easily be confused with lymphocytes or undifferentiated blasts, requiring confirmation by immunophenotyping (positive for CD41, CD42b, CD61) and small megakaryocyte enzyme labeling positivity.

### 6.3 Treatment selection

- TKI choices: First-generation TKI (imatinib) has limited efficacy in blast crisis, while second-generation TKI (dasatinib) shows an efficacy rate of <20% for the M7 subtype. The third-generation TKI (orelabatinib), with strong blood-brain barrier penetration and efficacy against T315I mutations, was selected as the first-line treatment for this case.
- Allogeneic hematopoietic stem cell transplantation: Due to the patient’s age and economic factors, transplantation was not performed immediately, and close follow-up is required subsequently.

### 6.4 Prognostic factors

The prognosis of M7 subtype in CML blast crisis is associated with the duration of CML before blast crisis, fusion gene type, and MRD level. In this case, BCR/ABL1 P210 fusion gene positivity and the decline in MRD during blast crisis suggest a potentially better prognosis than other subtypes.

## 7 Conclusion

This case of CML blast crisis (acute megakaryoblastic leukemia subtype) was diagnosed through multidisciplinary collaboration, suggesting:

- Splenomegaly and myelofibrosis should raise suspicion of CML blast crisis;
- Fusion gene and chromosomal karyotype testing are critical for diagnosis;
- The third-generation TKI orelabatinib may be effective for rare subtypes, though larger sample validation is needed.

## 8 Patient perspective

When I first noticed a mass in the left upper abdomen and my body grew increasingly weak, I was overwhelmed by fear and anxiety. The moment I was diagnosed with CML in blast crisis, it felt as though my world had collapsed. Learning that my condition was complex—not only a rare subtype of acute megakaryoblastic leukemia but also accompanied by multiple cytogenetic abnormalities and secondary myelofibrosis—left me almost hopeless.

However, the medical team never gave up on me. They carefully considered my financial situation and chose olverembatinib, a third-generation TKI, as the treatment plan. Every day during the initial treatment, I prayed for a turnaround. Two weeks later, the news that my bone marrow MRD had significantly decreased was indescribably joyous. This experience made me acutely aware that the doctors were not only highly professional but also deeply committed to fighting for the best possible treatment for me. Looking back, I still vividly recall the multidisciplinary team working together to diagnose my condition and repeatedly discussing the treatment plan. Their perseverance and unwavering support have given me the courage and confidence to keep battling this disease.

## Data Availability

The original contributions presented in the study are included in the article/supplementary material. Further inquiries can be directed to the corresponding authors.
